# The impact of psychological factors on condition-specific, generic and individualized patient reported outcomes in low back pain

**DOI:** 10.1186/s12955-017-0593-0

**Published:** 2017-02-21

**Authors:** Ida Løchting, Andrew M. Garratt, Kjersti Storheim, Erik L. Werner, Margreth Grotle

**Affiliations:** 1 Communication- and Research Unit for Musculoskeletal Disorders (FORMI), Oslo University Hospital & University of Oslo, Ullevaal, Nydalen, P.O. Box 4950, 0424 Oslo, Norway; 2 Department of Clinical Medicine, University of Oslo, Blindern, P.O. Box 1171, 0318 Oslo, Norway; 3 Knowledge Centre for the Health Services, Norwegian Institute of Public Health, Nydalen, Postbox 4404, 0403 Oslo, Norway; 4 Department of Health Sciences, University of Oslo, Blindern, P.O. Box 1074, 0316 Oslo, Norway; 5 Research Unit for General Practice, Uni Research, Uni Health, Bergen, Norway; 6 Oslo and Akershus University college, Faculty of Health Science, St. Olavs Plass, P.O. Box 4, N-0130 Oslo, Norway; 7 Faculty of Medicine, Institute of Health and Society, Department of General Practice, University of Oslo, Blindern, P.O. Box 1130, 0318 Oslo, Norway

**Keywords:** Low back pain, Patient reported outcomes, Illness perceptions, Individualized, Patient generated index, Psychological, Quality of life

## Abstract

**Background:**

An individualized patient reported outcome (PRO) has recently been recommended within LBP research, but no study has evaluated this instrument with commonly applied PROs. Moreover, the impact of psychological factors has mostly been assessed for disease-specific instruments. The objective of this study was to assess the predictive value of illness perceptions, pain catastrophizing and psychological distress on 12 month outcomes assessed by specific, generic and individualized PROs recommended in low back pain (LBP).

**Methods:**

Secondary analysis of patients with sub-acute or chronic LBP recruited for a cluster randomized controlled trial in primary care who completed a self-administered questionnaire at baseline and 12 months. 12 month scores for the Roland Morris Disability Questionnaire (RMDQ), the EuroQol (EQ-5D), and the Patient Generated Index (PGI) were dependent variables in hierarchical regression analysis. Independent variables included baseline scores for the Brief Illness Perceptions Questionnaire (Brief IPQ), Hopkins Symptom Check List (HSCL-25), Pain Catastrophizing Scale (PCS), health/clinical and sociodemographic variables.

**Results:**

Of the 216 eligible patients included, 203 patients responded to the baseline questionnaire and 150 (74%) responded at 12 months. The mean age was 38.3 (SD 10.2) years and 57.6% were female. The Brief IPQ showed a statistically significant variation in the 12-months score of all the PROs, explaining 2.5% in RMDQ, 7.9% in EQ-5D, and 3.6% in PGI. Most of the explained variation for EQ-5D scores related to illness perceptions. The PCS explained 3.7% of the RMDQ and 2.5% in the EQ-5D scores. The HSCL-25 did not make a significant contribution.

**Conclusion:**

Illness perceptions and pain catastrophizing were associated with 12-month outcomes as assessed by condition-specific, generic and individualized PROs. The Brief IPQ and PCS have relevance to applications in primary care that include interventions designed to enhance psychological aspects of health and where the contribution of such variables to outcomes is of interest. Further studies should assess whether the Brief IPQ perform similarly in LBP populations in other health care settings.

## Background

The identification of key prognostic factors is an important research area in low back pain (LBP) [1, 2]. This includes psychological factors which being modifiable, have relevance to clinical settings and guidelines recommend their assessment when treating patients [3, 4]. The most widely explored psychological factors are distress, fear avoidance beliefs and pain catastrophizing [5–7]. However, a recent systematic review evaluating which psychological factors might be associated in the transition from acute to chronic LBP in primary care, concluded that these psychological factors show modest predictive ability on outcomes including disability, pain and work status [6]. Illness perceptions is another psychological construct that has received increasing attention in back pain research but few independent prospective studies have assessed its role as a predictor of scores for patient reported outcomes (PROs) [8–11]. One study that compared the predictive ability of 20 psychological constructs in LBP, including fear avoidance beliefs, pain catastrophizing, and distress found that illness perceptions was the best predictor of disability at six months [9]. Aspects of illness perceptions also best predicted outcome at five years in this patient group [11].

The impact of psychological factors has largely focused on disease-specific PRO instruments including the Roland-Morris Disability Questionnaire (RMDQ) [5, 6]. Instruments assessing broader constructs including general health status and quality of life have received less attention, as reflected in their less frequent inclusion in reviews of the impact of psychological factors on LBP [5, 6]. Recent concerns have been raised that other types of outcomes which are important to back pain patients, are not part of the recommended outcome core set for back pain [12–14]. Sleep, social factors, fatigue and emotional well-being are areas found to be more important to patients with LBP than currently recognized [12–14]. This has led to the recommendation that an individualized measure of quality of life, the Patient Generated Index (PGI), should be included alongside the core set of outcome measures [13]. Individualized instruments allow the patient to select areas of importance to them. Hence their inclusion means that patients have the opportunity to include aspects of life that are not included in standardized instruments, but considered important in outcome assessment [15, 16]. PRO instruments that assess outcomes of importance to the individual patient complement clinical and health services evaluation and promote health outcomes more generally. It has also been argued that the absence of areas of less relevance reduces the level of ‘noise’ present in standardized instruments which can improve methodological aspects of outcomes, including responsiveness to change [17, 18].

The objective of this study was to assess the impact of illness perceptions, pain catastrophizing and psychological distress on recommended back pain PROs 12 months after an episode of non-specific LBP. The PROs included two of the most widely used instruments in patients with back pain, the RMDQ and the EQ-5D, and the recently recommended individualized PGI which has been validated in LBP [16].

## Methods

### Design and setting

This a-priori planned secondary analysis of a cluster randomized controlled trial [19, 20], included 220 patients recruited between 2008–2012 from general practitioners (GPs) and physiotherapists (PTs). The clinicians were randomly assigned to either provide a cognitive patient education intervention, based on the understanding of the physiology of pain or usual care. Treatment was provided once a week in one to one 30 min sessions for up to four consecutive weeks in the clinicians’ usual primary care settings, located in the greater Oslo area. We refer to the study protocol for further details on recruitment and treatment [19]. We followed recommendations and guidelines for reporting a multivariate prediction model where appropriate for this study [1, 21].

### Data collection

Clinicians recruited patients with non-specific LBP lasting 4–52 weeks, aged 20–55 years old with a score of ≥ 4 on the RMDQ. Exclusion criteria were possible nerve root pain or severe pathology, “red flags” or demand for a specific treatment. Following informed consent, the baseline questionnaire was completed in the provider’s waiting room or at home. Patients received a 12 month follow-up postal questionnaire.

The study was approved by the Norwegian Regional Committee for Medical Research Ethics East and the Data Inspectorate and followed the Helsinki Declaration.

### Outcomes and psychological instruments

The self-completed questionnaire included the PRO and psychological instruments, numerical rating scale of back pain (0–10) and questions relating to age, sex, ethnicity (born in Norway- yes/no), education (≤12 years/> 12 years), employment (employed/student/unpaid work or unemployed/rehabilitation/sickleave), smoking (yes/no), co-morbidity (yes/no), BMI, previous LBP (yes/no).

PRO instruments included disability in daily activities assessed by the RMDQ, which has 24 yes/no items that sum to a score from 0–24, where 24 is the most severe back related disability [22]. The RMDQ has evidence for data quality, reliability and validity in Norwegian LBP patients [23].

General health status was assessed by the EQ-5D [24] 3-L version which is a utility instrument with five items that have three-point descriptive scales of no problem, some problems and severe problems. The EQ-5D index is based on utility weights from the general population and is scored from −0.59 to 1.0 were 1 is the best possible score. The EQ-5D has evidence for reliability and validity in Norwegian LBP patients [25].

Individual quality of life was assessed by the PGI completed in three stages [16, 26]. In the first stage, patients list up to five important areas in their life affected by their LBP. In the second, they rate the extent to which their LBP has affected them in each area and in the rest of their lives on a seven point scale from “the worst they can imagine” to “exactly as they would like it to be”. In the third stage they are asked to imagine that they can improve some or all of the areas with ten points to distribute across the areas that they would most like to improve. The PGI score from 0–100 represents the worst and best possible quality of life respectively. The PGI has evidence for reliability and validity in Norwegian patients with LBP [16].

Three psychological instruments were included as predictors. Illness perceptions was assessed by the Brief Illness Perceptions Questionnaire (Brief IPQ) [27] which has nine items comprising cognitive and emotional illness perceptions. The items have a 0–10 scale with endpoint descriptors. The items sum to give a score from 0 to 100 with higher scores representing a more threatening view of illness. The instrument has evidence for reliability and validity in Norway [28].

Psychological distress was assessed by the Hopkin’s Symptom Check List (HSCL-25), which has 25 items [29]. The HSCL-25 asks about symptoms during the last week and items have a four-point scale from “not at all” to “to a large extent”. Items sum to give a score from 0 to 4 where 4 is the most severe symptoms. The Norwegian HSCL-25 version has been used in several studies, including patients with LBP [30–32], however evidence relating to reliability and validity in patients with LBP is lacking.

Pain catastrophizing was assessed by the 13-item Pain Catastrophizing Scale (PCS) [33] which asks about past painful experiences and the degree to which they experienced this on a five-point scale from “not at all” to “all the time” (31). Items sum to give a score from 0 to 52 where 52 is the highest level of catastrophizing. The PCS has evidence for reliability and validity in Norwegian patients with LBP [34].

### Statistical analysis

Hierarchical multiple regression analysis [35] was used to assess the contribution of baseline Brief IPQ, HSCL-25 and PCS scores to those for the 12 months RMDQ, EQ-5D and PGI scores, after controlling for baseline sociodemographic and health/clinical variables. Univariate analysis informed variable selection and hence the three models differ slightly. Variables that made a significant contribution at the *p* < 0.10 level to the dependent variables were included in the final multivariate regression analysis in addition to sex, age, type of treatment and type of clinician. Sample size requirements took into account the number of independent variables used (*N* > 50+ 8 m) [36]. The assumptions of regression analysis were assessed including normality, presence of outliers and no multicollinearity. Normal probability plots were examined and the correlation between the independent variables assessed [36]. Sociodemographic variables were entered in the first step, health status and clinical variables in the second, and each of the psychological instruments was entered in the third to fifth steps. PGI scores at follow-up are usually based on stage three points at follow-up administration. However, for purposes of intervention studies it is recommended that baseline points are used which reflect the patients original priorities for improvement [34]. Therefore, a sensitivity analysis was conducted with this alternative method of scoring the PGI also as dependent variable. The 5% significance level was used.

SPSS version 21 was used for all the statistical analysis.

## Results

### Data collection

Of the 220 patients, four were excluded because they were retrospectively found not to meet the inclusion criteria. Of the 216 eligible patients, 203 patients responded to the baseline questionnaire and 150 (74%) at 12 months. The most frequent reason for loss at follow-up was lack of response to reminders by mail or telephone. Compared to responders, non- responders at 12 months had higher BMI (*p* < 0.01), a lower education level (*p* < 0.05) and a greater proportion received treatment from GPs compared to PTs (*p* < 0.05).

Table 1 shows the patients sociodemographic characteristics and mean scores for the PRO instruments. Table 2 shows the univariate analysis of the contribution of baseline variables to the three types of PROs at 12 months. There was no deviation from normality, evidence of outliers or multicollinearity in the multivariate analyses. Table 3 shows the results of the hierarchical multiple regressions analyses with the RMDQ as dependent variable at 12 months. Two of the three psychological instruments significantly contributed to explaining variation in RMDQ scores separately; 2.5% and 3.7% for the Brief IPQ and PCS respectively (Table 3, models 3–5). Health /clinical variables explained 12.2% of the variation (model 2). The model that explained the highest percentage of variation at 25.6% included the PCS, health/clinical and sociodemographic variables.

**Table 1:** Patient characteristics for patients responding to the baseline questionnaire (*n* = 203)

Variables	
Independent variables	
Sex (n, %)	
Male	86 (42.4)
Female	117 (57.6)
Age years (mean, SD)	38.3 (10.2)
Born in Norway (n,%)	
Yes	171 (84.7)
No	31 (15.3)
Clinical setting (n,%)	
Medical doctor	62 (30.5)
Physiotherapist	141 (69.5)
Type of treatment (n, %)	
Usual care	100 (49.3)
Cognitive intervention	103 (50.7)
Education level	
12 years or less	54 (26.7)
> 12 years	148 (73.3)
Employment status (n, %)	
Employed/student	122 (60.1)
Unemployed/rehab/sickleave	81 (39.9)
Smoking	
Yes	21 (10.3)
No	182 (89.7)
BMI (mean, SD)	25.5 (4.2)
Previous LBP (n,%)	
Yes	173 (85.6)
No	29 (14.4)
Co-morbidity (n,%)	
Yes	111 (55.5)
No	89 (44.5)
Back pain, NRS (0–10)	5.1 (2.1)
Brief-IPQ (mean, SD)^a^	52.5 (12.4)
PCS (mean, SD)^b^	15.9 (9.2)
HSCL-25 (mean, SD)^c^	1.6 (0.4)
Outcome variables	
RMDQ (mean, SD)^d^	9.3 (4.0)
EQ-5D (mean, SD)^e^	0.6 (0.3)
PGI (mean, SD)^f^	38.4 (14.8)

^a^Brief-IPQ (0–100); higher scores represent a more threatening view of the illness

^b^PCS (0–52); higher scores represent higher levels of catastrophizing

^c^HSCL-25 (1–4); higher scores represent more severe symptoms

^d^RMDQ (0–24); higher scores represent greater overall disability

^e^EQ-5D (−0.59 to 1.0); higher scores represent better health status

^f^PGI (0–100); higher scores represent better quality of life

**Table 2:** Univariate analysis of the contribution of baseline variables to three types of patient-reported outcomes; the diseases-specific RMDQ, the generic EQ-5D scores and the individualized PGI at 12 months

Variables	RMDQ^e^ 12 m			EQ-5D^f^ 12 m			PGI^g^ 12 m		
	(*n* = 147)			(*n* = 150)			(*n* = 137)		
	R2	β^a^	*p*- value	R2	β ^a^	*p*- value	R2	β^a^	*p*- value
Sociodemographic									
Sex (wome*n* = 2)	.004	-.506	.433	.000	-.005	.912	.005	3.222	.421
Age years	.000	-.002	.940	.000	-.001	.791	.000	.004	.982
Ethnicity (Norwegian) (yes/no) (no = 2)	.040	2.118	.016	.023	-.105	.066	.009	−6.148	.264
Education level (>12 years = 2)	.046	−1.863	.009	.049	.126	.006	.015	6.453	.148
Employment status (employed/ unemployed) (unemployed =2)	.017	1.023	.115	.021	-.073	.079	.006	3.724	.356
Smoking (yes/no) (yes = 2)	.020	1.780	.088	.014	-.098	.145	.004	−4.476	.491
Health/clinical									
BMI	.017	.121	.116	.009	-.006	.250	.016	-.696	.143
Previous LBP (yes/no) (yes = 2)	.005	.764	.401	.002	-.035	.554	.000	-.224	.968
Back pain (NRS) (0–10)	.042	.382	.013	.034	-.022	.024	.023	−1.711	.076
Co-morbidity (yes/no) (yes = 2)	.000	.136	.833	.000	-.004	.928	.007	−3.789	.344
Clinical setting (Medical Doctor/ Physio) (MD = 2)	.085	2.402	.000	.053	-.123	.005	.014	−5.915	.162
Treatment (usual care/cognitive) (usual care = 2)	.008	-.697	.274	.002	.024	.552	.002	1.878	.635
Psychological									
Brief-IPQ^b^ (0–100)	.146	.118	.000	.185	-.009	.000	.068	-.483	.002
HSCL-25^c^ (1–4)	.055	2.065	.004	.062	-.143	.002	.025	−8.419	.063
PCS^d^ (0–52)	.148	.162	.000	.097	-.009	.000	.033	-.456	.034
Outcome variables									
RMDQ^e^ baseline (0–24)	.097	.301	.000	.060	-.015	.003	.004	-.386	.437
EQ-5D^f^ baseline (−0.59-1)	.097	−4.536	.000	.082	.272	.000	.021	12.589	.092
PGI^g^ baseline (0–100)	.013	-.030	.182	.006	.001	.391	.007	.134	.336

^a^Unstandardized beta

^b^Brief-IPQ; higher scores represent a more threatening view of the illness

^c^PCS; higher scores represent higher levels of catastrophizing

^d^HSCL-25; higher scores represent more severe symptoms

^e^RMDQ; higher scores represent greater overall disability

^f^EQ-5D; higher scores represent better health status

^g^PGI; higher scores represent better quality of life

**Table 3:** Final hierarchical linear regression analysis of the relationship between RMDQ on the psychological instrument scores, health, clinical and sociodemographic variables (*n* = 147)

	R^2^	R^2^ Change	β ^a^	95% CI for B	St. β	*p*-value
Variables						
Model 1: sociodemographic^b^	9.7%	9.7%				
Model 2: health/clinical^c^	21.9%	12.2%				
Model 3: illness perceptions^d^	24.4%	2.5%				
Brief-IPQ (0–100)			.060	.004 to.117	.195	.035
Model 4: pain catastrophizing^e^	25.6%	3.7%				
PCS (0–52)			.094	.022 to.167	.224	.011
Model 5: psychological distress^f^	22.0%	0.1%				
HSCL-25 (1–4)			.307	−1.305 to1.919	.035	.707

^a^Unstandardized Beta

^b^Model 1: sex, age, ethnicity, education and smoking

^c^Model 2: back pain (NRS), baseline RMDQ, baseline EQ-5D, clinical setting (GP or PT) and type of treatment (usual care or cognitive intervention) in addition to the sociodemographic variables (model 1)

^d^Model 3: illness perceptions by the Brief IPQ (sum score) in addition to the variables in model 1 and 2

^e^Model 4: pain catastrophizing by the PCS (sum score) in addition to the variables in model 1 and 2

^f^Model 5:) psychological distress by the HSCL-25 (sum score) in addition to the variables in model 1 and 2

The PCS and the Brief IPQ also made significant contributions to explaining variation in EQ-5D scores separately; 2.5% for the PCS and 7.9% for the Brief IPQ (Table 4, models 3–5). Independently, Brief IPQ scores (7.9%) explained about the same amount of variation in EQ-5D as the sociodemographic (7.5%) and health/clinical (7.2%) variables. The model that explained the highest percentage of variation at 22.6% included the Brief IPQ, health/ clinical and sociodemographic variables.

**Table 4:** Final hierarchical linear regression analysis of the relationship between EQ-5D and the psychological scales adjusted for sociodemographic and health/clinical variables (*n* = 150)

	R^2^	R^2^ change	β ^a^	95% CI for B	St. β	p-value
Variables						
Model 1: sociodemographic^b^	7.5%	7.5%				
Model 2: health/clinical^c^	14.7%	7.2%				
Model 3: illness perceptions^d^	22.6%	7.9%				
Brief-IPQ (0–100)			-.007	-.011 to -.003	-.345	.000
Model 4: pain catastrophizing^e^	17.2%	2.5%				
PCS (0–52)			-.005	-.010 to.000	-.184	.045
Model 5: psychological distress^f^	15.3%	0.6%				
HSCL-25 (1–4)			-.056	-.166 to.054	-.098	.316

^a^ Unstandardized Beta

^b^Model 1: sex, age, ethnicity, education and employment

^c^Model 2: back pain (NRS 1–10), baseline RMDQ, baseline EQ-5D, clinical setting (GP or PT) and type of treatment (usual care or cognitive intervention) in addition to the sociodemographic variables in model 1

^d^Model 3: illness perceptions by the Brief IPQ (sum score) in addition to the variables in model 1 and 2

^e^Model 4: pain catastrophizing by the PCS (sum score) in addition to the variables in model 1 and 2

^f^Model 5:) psychological distress by the HSCL-25 (sum score) in addition to the variables in model 1 and 2

The Brief IPQ was the only psychological instrument that explained a statistically significant component (3.6%) of the variation in the PGI, which was similar to that for the health status and clinical variables (Table 5). The model that explained the highest percentage of variation at 8.1% included the Brief IPQ, health/clinical and sociodemographic variables. The use of PGI baseline stage three points did not affect the results (not shown).

**Table 5:** Final hierarchical linear regression analysis of the relationship between PGI and the psychological scales adjusted for sociodemographic and health/clinical variables (*n* = 137)

	R^2^	R^2^ Change	β^a^	95% CI for B	St. β	*p*-value
Variables						
Model 1: sociodemographic^b^	0.5%	0.5%				
Model 2: health/clinical^c^	4.6%	4.1%				
Model 3: illness perceptions^d^	8.1%	3.6%				
Brief-IPQ (0–100)			-.426	-.819 to -.032	-.230	.034
Model 4: pain catastrophizing^e^	5.9%	1.3%				
PCS (0–52)			-.325	-.829 to .180	-.129	.205
Model 5: psychological distress^f^	5.6%	1.1%				
HSCL-25 (1–4)			−6.306	−17.126 to 4.514	-.119	.138

^a^Unstandardized Beta

^b^Model 1: sex, age

^c^Model 2: back pain, baseline PGI, baseline EQ-5D, clinical setting (GP or PT) and type of treatment (usual care or cognitive intervention) in addition to the sociodemographic variables (model 1)

^d^Model 3: illness perceptions by the Brief IPQ (sum score) in addition to the variables in model 1 and 2

^e^Model 4: pain catastrophizing by the PCS (sum score) in addition to the variables in model 1 and 2

^f^Model 5:) psychological distress by the HSCL-25 (sum score) in addition to the variables in model 1 and 2

The HSCL-25 did not make a significant contribution in any of the multivariate analyses (Tables 3, 4 and 5).

## Discussion

The aim of this study was to assess the impact of illness perceptions, pain catastrophizing and psychological distress on PROs recommended for back pain 12 months after an episode of non-specific LBP. Illness perceptions at baseline predicted 12 months scores for the three types of PROs: the disease-specific RMDQ, the generic EQ-5D and the individualised PGI. Pain catastrophizing predicted 12 months RMDQ and EQ-5D scores, but not those for the PGI. Finally, psychological distress did not show any significant association with any of the 12 month PRO scores. In prognostic LBP studies, baseline factors typically account for around 30% of the variation in outcome [37], with psychological aspects, explaining less than 5% in disease-specific outcomes including the RMDQ and the Oswestry Disability Index (ODI) [9, 10, 30]. The current findings for the impact of pain catastrophizing and illness perceptions on the RMDQ are in agreement with this literature.

The strongest association in the current study was between illness perceptions and 12-months scores of EQ-5D. Illness perceptions explained nearly 8% of the variation in 12-months EQ-5D. Moreover, illness perceptions was the only psychological factor that showed a significant impact on health-related quality of life, as assessed by the individualized PGI (3.6%). There are few published studies, which have evaluated the impact of illness perceptions on quality of life instruments. With the exception of one study that included the Short Form Health Survey (SF-36) as an outcome measure (10) and found that illness perceptions explained up to 14% of the variance, the proportion of variance explained by illness perceptions in the current study as assessed by the EQ-5D (8%) is greater than previously reported [9]. Our results are in agreement with previous studies showing that illness perceptions have been found to be associated with health outcomes of disability, pain and general health [9, 10]. Minor differences might be explained by the fact that the current study included the Brief IPQ with a sum scale of illness perceptions, whereas the other studies in LBP have used longer versions, without a sum scale [38]. The greater proportion of variation explained by illness perceptions when the EQ-5D was the dependent variable might be due to the broader focus of the EQ-5D as compared to the RMDQ with its focus on back-related disability. It would be interesting to consider the role of illness perceptions and pain catastrophizing in explaining other back-related and generic PROs.

Baseline PCS scores were significantly associated with disability as assessed by the RMDQ (3.7%) and general health as assessed by the EQ-5D (2.5%) at 12 months. A recent systematic review evaluating catastrophizing as a prognostic factor in LBP concluded that there is some evidence that catastrophizing as a coping strategy might lead to delayed recovery but that the influence of catastrophizing in patients with LBP is not fully established [39].

Distress did not explain any significant variation in the outcome measures included in our study. Previous systematic reviews that included psychological factors as predictors of outcomes in prospective cohorts of patients with LBP or musculoskeletal pain, point to an association between distress and PROs [5, 40]. The studies included in the reviews had outcomes relating to symptom satisfaction, the RMDQ and pain. A recent review found evidence for the predictive ability of distress in two of seven studies in primary care [6]. Different measures used to assess these concepts as well as different patient populations and PRO instruments may explain the variation across studies.

The independent variables included in the current study reflect the bio-psycho-social model where potentially important sociodemographic, health and clinical variables were included in the univariate analysis. The variables included in the final model explained between 22.6% and 25.6% for the EQ-5D and RMDQ respectively, which is consistent with other prospective studies [37]. There are no published studies that have examined the association between the independent variables included in this study and individualised outcomes as assessed by instruments such as the PGI. In the current study, only 5 of the 18 variables included in the univariate analysis were significantly associated with PGI scores at the 10% level and only illness perceptions explained statistically significant variation at the 5% level in the final model. The final model only explained 8.1% of the variation. Different options are available for administering the PGI at baseline and follow-up. The current study followed the latest recommendations by one of the developers including a closed format for the areas in stage one [41]. Following recent recommendations [41], a sensitivity analysis was conducted with the points given in stage three at baseline included at follow-up. The sensitivity analysis did not influence the results. The reliability and validity of the instruments included as dependent and independent variables places an upper limit on the size of the associations expected in both the current and previous studies. The Brief IPQ and PCS have evidence for these measurement properties in this group of patients [28, 34], but evidence is lacking for the HSCL-25 in Norwegian back pain patients. However, these considerations are relatively minor given the relatively low level of associations expected based on existing research of psychological prognostic factors [9, 10, 30].

### Study strengths and limitations

Study strengths include the a-priori secondary analysis of a cluster RCT with the inclusion of the three main types of PROs – specific, generic and individualized – recommended for use in LBP research. It has been argued that prognostic variables should be evaluated in patients who have received the same treatment or have been participants in a randomized trial [42]. The inclusion of both sociodemographic, health/clinical and psychological variables reflects the biopsychosocial model.

Respondent burden meant that only a selection of self-completed instruments assessing psychological aspects and outcomes could be included. The aims of the intervention study informed instrument selection including primary and secondary outcomes [19]. Instruments assessing other psychological aspects that are relevant to back pain patients including coping, fear avoidance beliefs and self-efficacy were not included. Moreover, the prediction models in this study were the ones that best suited our data, hence testing and validating the predictive performance in other samples is necessary [43]. Future studies should evaluate the psychometric properties of the HSCL-25 in patients with LBP. Finally, there was a loss to follow up of approximately 25%, which follows previous research in back pain [5, 6].

## Conclusions

Illness perceptions and pain catastrophizing predicted 12-months PRO scores including those for two of the most widely applied specific and generic instruments, the RMDQ and EQ-5D, as well as individualized outcomes as assessed by the PGI. The latter has had considerably less application but is recommended for back pain research as a means of addressing the limitations of current PRO instruments [13]. Differences were found for the type of outcomes and the amount of variation explained by the psychological instrument scores with illness perceptions making the largest overall contribution. Further studies are needed to assess whether these findings are replicated in other groups of patients with LBP. Future studies should consider the inclusion of illness perceptions as a potential determinant of health outcome as assessed by recommended PRO instruments in addition to more traditional psychological aspects.

## Abbreviations

Brief IPQ: The brief illness perceptions questionnaire
EQ-5D: The EuroQol
GPs: General practitioners
HSCL-25: The Hopkin’s symptom check list
LBP: Low back pain
PCS: Pain catastrophizing scale
PGI: Patient generated index
PROs: Patient-reported outcomes
PTs: Physiotherapists
RMDQ: Roland Morris disability questionnaire
SD: Standard deviations
SF-36: Short form health survey
